# A novel visceral adiposity index predicts bone loss in female early rheumatoid arthritis patients detected by HR-pQCT

**DOI:** 10.1038/s41598-023-29505-z

**Published:** 2023-02-11

**Authors:** Jiang Yue, Priscilla C. H. Wong, Ying Zhang, Feng Peng, James F. Griffith, Jiankun Xu, Fan Xiao, Tena K. Li, Vivian Hung, Ling Qin, Lai-Shan Tam

**Affiliations:** 1 Department of Endocrinology and Metabolism, Ren Ji Hospital, School of Medicine, Shanghai Jiao Tong University, Shanghai, China; 2 Department of Medicine & Therapeutics, The Prince of Wales Hospital, The Chinese University of Hong Kong, Hong Kong, China; 3 Department of Laboratory Medicine, Ren Ji Hospital, School of Medicine, Shanghai Jiao Tong University, Shanghai, China; 4 Department of Imaging and Interventional Radiology, The Prince of Wales Hospital, The Chinese University of Hong Kong, Hong Kong, China; 5 Bone Quality and Health Center, Department of Orthopedics & Traumatology, The Prince of Wales Hospital, The Chinese University of Hong Kong, Hong Kong, China

**Keywords:** Diseases, Rheumatic diseases, Musculoskeletal abnormalities, Rheumatoid arthritis

## Abstract

The purpose of this prospective study is to compare the Chinese visceral adiposity index (CVAI) between early rheumatoid arthritis (ERA) patients and healthy controls; and to assess the relationship between CVAI and the bone microstructure using high-resolution peripheral quantitative computed tomography (HR-pQCT) in ERA patients. 104 female ERA and 100 age-, gender- and BMI-matched healthy controls were recruited for the comparison of CVAI. All ERA patients were prospectively followed for 1 year. HR-pQCT scan of the distal radius, tibia and second metacarpal head were performed at baseline and after one-year. ERA patients were divided into two sub-groups according to the median CVAI value (65.73) (low CVAI and high CVAI groups). CVAI in the ERA group was significantly higher than the controls group (*p* = 0.01). At baseline, the high CVAI group had a higher ESR level (*p* = 0.004) while the cortical volumetric bone mineral density (vBMD) was lower (at both the distal radius and tibia, all *p* < 0.05) compared to the low CVAI group. Linear regression analysis revealed that a higher baseline CVAI was an independent predictor of a lower cortical vBMD at month 12 (distal radius: B = − 0.626, p = 0.022, 95%CI − 1.914  to  − 0.153; tibia: B = − 0.394, p = 0.003, 95%CI − 1.366 to − 0.290); and a greater reduction in trabecular vBMD (tibia: B = 0.444, p = 0.001, 95%CI 0.018–0.063; distal radius: B = 0.356, p = 0.008, 95%CI 0.403–0.063). In summary, CVAI is an independent predictor of trabecular bone loss in female patients with ERA, which may be augmented by a chronic inflammatory state in patients with visceral dysfunction of fat metabolism.

**Trial registration:** http://Clinicaltrial.gov no: NCT01768923, 16/01/2013.

## Introduction

Rheumatoid arthritis (RA) is a chronic, systemic inflammatory autoimmune disease that primarily attack joints leading to progressive joint destruction^1^. It is characterized by synovial hyperplasia, immune cell activation, articular inflammation, invasion of synovium to adjacent bone and cartilage resulting in bone erosion^1^. Excessive production of pro-inflammatory cytokines trigger bone resorption by stimulating osteoclasts directly and by inhibiting osteoblast function, resulting in systemic bone loss manifested as osteoporosis diagnosed by a low bone mineral density (BMD) measured using dual-energy x-ray absorptiometry (DXA)^2^. Using high-resolution peripheral quantitative computed tomography (HR-pQCT), our previous studies showed that intra-articular bone loss at the metacarpal head and periarticular bone loss at the distal radius and tibia have also been demonstrated in these patients^3,4^.

Obesity has been traditionally considered to be protective against osteoporosis and fracture. Adipose tissue is no longer thought of as solely a triglyceride storage compartment and neighboring tissue insulator, but rather as an active endocrine organ with multiple functions^5^. Dyslipidemia appears to manifest in RA patients in both early and advanced disease. White adipose tissue (WAT) is recognized as an important endocrine organ and regulates metabolism by affecting other organs, such as bone and immune system^6^. Visceral adipose tissue (VAT) acts as an active as well as metabolic organ. It secretes inflammatory matters^7^ and adipokines^8^. Meanwhile, it also plays a critical role in metabolizing steroid hormones^9^ that affect skeletal metabolism. VAT has been related to a low BMD and been a significant risk factor for some types of fractures^10^. Computed tomography (CT) and magnetic resonance imaging (MRI) are applied for quantitatively measuring the VAT, but these imaging techniques are expensive and not routinely assessed^10^. Recently, a clinical visceral adiposity index (VAI) calculated from waist circumference (WC), body mass index (BMI), serum triglycerides (TG), and high-density lipoprotein (HDL) levels, was performed to quantitatively assess the visceral adiposity^11^. VAI was significantly associated with a number of metabolic diseases, such as cardiovascular and cerebrovascular disease^11^, metabolic fatty liver disease (MAFLD)/non-alcoholic steatohepatitis (NASH)^12^, polycystic ovary syndrome in western countries^13,14^. However, VAI was poorly associated with adipose tissue area in Chinese (AUROC: 0.69[0.65–0.73], *P* < 0.001)^15^ and striking differences in terms of body fat allocation exist between different ethnicities. Asians are featured by a comparatively higher body fat content at lower BMI variables comparing to western population and are more prone to visceral fat accumulation^16^. Chinese visceral adiposity index (CVAI), including age, BMI, WC, TG and HDL-C, is a clinical confirmed index for the assessment of visceral fat dysfunction. Age is the only factor that is different between VAI and CVAI. In a study involving 6495 Chinese subjects, CVAI was related to visceral obesity and insulin resistance; and was able to discriminate patients with and without metabolic syndrome, hypertension, diabetes and prediabetes better than BMI and WC. The AUROC of CVAI for visceral obesity was 0.83, which was significantly higher than VAI (0.69)^15^.

A recent HR-pQCT study demonstrated that higher VAT was associated with greater BMD and better microstructure of the peripheral skeleton despite observation on deleterious changes in the cortical compartment of the non–weight bearing radius^10^. Associations were no longer significant after adjustment for BMI or weight, suggesting that VAT might not have a substantial effect on the skeleton metabolism independent of BMI or mechanical loading^10^. Nonetheless, in patients with RA, moderate inflammatory activity was associated with greater visceral adipose tissue^17^. Whether visceral adiposity measured using CVAI in patients with RA may predict accelerated intra-articular and periarticular bone loss in patients remains for our exploration.

## Results

### Characteristics of participants in this study

Table 1 demonstrated the characteristics of the patients (at baseline) and controls. ERA patients and healthy controls were comparable in terms of age, body mass index (BMI), serum triglycerides (TG), and HDL-c levels, visceral adiposity index (VAI) and the prevalence of overweight/obesity and visceral obesity. Nevertheless, waist circumference (WC), Chinese visceral adiposity index (CVAI), erythrocyte sedimentation rate (ESR) and C- response protein (CRP) levels were significantly higher in ERA patients compared to healthy controls (*p* < 0.05, Table 1).

**Table 1:** Baseline characteristics of participants.

	Healthy control (n = 100)	Female ERA (n = 104)	*P *value
Age (years)	46 ± 6	48 ± 11	0.291
Height (cm)	161 ± 5	160 ± 8	0.343
Weight (kg)	59.4 ± 14.2	57.4 ± 9.4	0.150
BMI (kg/m^2^)	20.6 ± 2.4	21.4 ± 2.5	0.109
WC (cm)	70.7 ± 6.7	78.9 ± 6.7	0.002
HDL-C (mmol/L)	1.61 ± 0.52	1.46 ± 0.40	0.497
TG (mmol/L)	0.95 ± 0.56	1.02 ± 0.47	0.317
CVAI	41.12 ± 20.89	55.10 ± 32.02	0.010
VAI	63.75 ± 59.74	65.72 ± 38.67	0.870
Overweight/obesity	26 (26.0%)	28 (26.9%)	1.000
Visceral obesity	20 (20%)	31 (29.8%)	0.145
ESR (mm/h)	17.8 ± 2.3	64.1 ± 32.0	0.000
CRP (mg/L)	1.1 ± 0.6	20.9 ± 26.8	0.000
Disease duration (month)	NA	7.2 ± 6.3	
HAQ (0–3)	NA	1.1 ± 0.7	
Tender joint count (0–68)	NA	8 (5–13)	
Swollen joint count (0–66)	NA	5 (3–7)	
Damage joint count (0–68)	NA	2 (1–4)	
DAS 28-CRP	NA	5.0 ± 1.0	
DAS 28-ESR	NA	6.9 ± 1.1	
SDAI score	NA	29.7 ± 11.6	
Biologic DMARDs	NA	0	
MTX	NA	9	
Leflunomide	NA	1	
Sulfasalazine	NA	2	
Hydroxychloroquine	NA	6	
Glucocorticord	NA	19	
NSAIDs use	NA	82	

BMI: body mass index; WC: waist circumference; FBG: fasting blood glucose; LDL: low density lipoprotein; HDL: high density lipoprotein; TC: total cholesterol; TG: triglyceride; CVAI: Chinses visceral adiposity index; VAI: visceral adiposity index; ESR: erythrocyte sedimentation rate; CRP: C- response protein; HAQ: Health Assessment Questionnaire; DAS28: Disease Activity Score in 28 joints; SDAI: Simple Disease Activity Index Score; DMARD, disease modifying anti-rheumatic drugs; MTX, Methotrexate; NSAIDs, non-steroidal anti-inflammatory drugs. *P* value: healthy control & ERA.

### The association between CVAI and clinical parameters of RA patients at baseline

The HR-pQCT parameters at baseline and month 12 are summarized in supplementary Table 1. At baseline, CVAI had a low to moderate correlation with clinical markers of disease severity [damage joint counts (r = 0.272, *p* = 0.005), ESR (r = 0.239, *p* = 0.015), rheumatoid factor (RF) (r = 0.257, *p* = 0.008) and anti‐cyclic citrullinated peptide (ACCP) titer (r = 0.220, *p* = 0.025)].

In terms of HR-pQCT parameters, significant changes over the 12 months existed in cortical porosity diameter (Ct. Pm) of distal tibia (97.27 ± 7.43 and 95.52 ± 6.37, *p* = 0.021) and compact BMD (Dcomp) (886.10 ± 69.05 and 905.94 ± 60.20, *p* = 0.012), Ct. Pm (62.57 ± 6.53 and 59.49 ± 8.48, *p* = 0.011), meta trabecular BMD (Dmeta) (186.95 ± 44.61 and 173.46 ± 49.73, *p* = 0.047) of distal tibia in female ERA patients (Supplementary Table 1).

### HR-pQCT parameters in the two CVAI subgroups

ERA patients were then divided into two subgroups according to the median of CVAI (65.73): low CVAI group (< 65.73) and high CVAI group (≥ 65.73). With regards to the clinical features, some markers of disease severity (including the number of damaged joints, ESR level, RF titer and health assessment questionnaire (HAQ)) were higher in the high CVAI group than the low CVAI group (all* p* < 0.05, Table 2). CRP, disease duration, ACCP titer, tender joint count, swollen joint count, disease Activity Score in 28 joints (DAS28)-CRP, simple disease activity index (SDAI) score and patient visual analogue scale (VAS) global assessment were similar between the two groups (*p* > 0.05, Table 2).

**Table 2:** Clinical characteristics of female ERA subgroups divided according to the median of CVAI (65.73).

	Low CVAI (n = 52)	High CVAI (n = 52)	*P* value
ESR (mm/h)	55.52 ± 27.52	73.63 ± 34.49	0.004
CRP (mg/L)	18.09 ± 21.09	24.58 ± 31.76	0.222
Disease duration (month)	8.0 ± 5.0	9.0 ± 7.0	0.414
RF titer	0.69 ± 0.47	0.92 ± 0.27	0.003
ACCP titer	0.92 ± 0.27	0.81 ± 0.40	0.087
HAQ (0–3)	0.93 ± 0.63	1.31 ± 0.77	0.007
Tender joint count (0–68)	8 (5.12)	8 (5.13)	0.601
Swollen joint count (0–66)	6 (2.8)	4 (36.75)	0.216
Damage joint count (0–68)	1.5 (0.252)	2 (1.4)	0.026
DAS 28-CRP	4.94 ± 1.08	5.06 ± 0.85	0.536
SDAI score	29.87 ± 12.80	29.64 ± 10.54	0.921
Patient VAS global assessment	6.34 ± 1.82	6.19 ± 1.72	0.678
Biologic DMARDs	0	0	–
MTX (%)	6 (11.54)	3 (5.77)	0.319
Leflunomide (%)	1 (1.92)	0	1.000
Sulfasalazine (%)	1(1.92)	1(1.92)	–
Hydroxychloroquine (%)	4 (7.69)	2 (3.85)	0.402
Glucocorticord (%)	12 (23.08)	7 (13.46)	0.310
NSAIDs use (%)	43 (82.69)	39 (75)	0.472

CVAI: Chinses visceral adiposity index; low CVAI group (< 65.73); high CVAI group (≥ 65.73). ERA: early rheumatoid arthritis; ESR: erythrocyte sedimentation rate; CRP: C- response protein; RF: rheumatoid factor; ACCP: Autoantibodies binding to citrullinated antigens; HAQ: Health Assessment Questionnaire; DAS 28-CRP: Disease Activity Score in 28 joint for CRP; CRP: C- response protein; SDAI: Simple Disease Activity Index Score; VAS: visual analogue scale. NSAIDs: non-steroidal anti-inflammatory drugs. *P* value: low CVAI & high CVAI.

At baseline, cortical porosity diameter at the metacarpal head (MC) 2 was larger while the cortical volumetric bone mineral density (vBMD) at the tibia and distal radius were significantly lower in the high CVAI group compared to the low CVAI group (all *p* < 0.05, Supplementary Tables 2–4). No significant differences existed between the two groups in terms of trabecular bone density, trabecular thickness (Tb. Th), cortical thickness (C.Th) and trabecular bone volume fraction (all *p* > 0.05, Supplementary Tables 2–4).

The changes in cortical vBMD at the distal radius and trabecular vBMD at the tibia after 12 months were greater in the high CVAI group compared to the low CVAI group (distal radius: 15.11 ± 2.96 and 11.73 ± 2.30, *p* = 0.039; distal tibia: 4.41 ± 0.83 and 2.60 ± 0.51, *p* = 0.032). No significant differences existed between the two groups in terms of other changes in HR-pQCT parameters after 12 months (all *p* > 0.05, data not shown).

Significant changes over the 12 months existed in the changed average BMD (10.19 ± 60.86 and − 6.33 ± 30.49, *p* = 0.042), changed compact BMD (19.28 ± 57.09 and − 15.11 ± 42.76, *p* = 0.006), changed Ct.Th (0.046 ± 0.22 and − 0.046 ± 0.14, *p* = 0.016) of distal tibia and changed compact BMD (39.04 ± 61.94 and − 62.36 ± 241.56, *p* = 0.014), changed average BMD (19.20 ± 78.20 and − 37.48 ± 91.25, *p* = 0.036), changed Dtrab (− 10.24 ± 60.93 and − 19.30 ± 38.85, *p* = 0.014), changed Dmeta (− 14.99 ± 64.15 and − 23.61 ± 49.02, *p* = 0.036) as well as changed Dinn (− 7.08 ± 60.17 and − 16.33 ± 35.24, *p* = 0.035) of distal radius between low CVAI and high CVAI groups (Supplementary Tables 3, 4).

### Association between baseline CVAI and bone microstructure detected by HR-pQCT at month 12

Linear regression analysis was performed to ascertain whether baseline CVAI was an independent predictor of bone microstructure at month 12. Other potential explanatory variables at baseline included demographic characteristic (age, BMI), disease-specific parameters (RF, ACCP, disease duration) as well as treatment. Linear regression analysis revealed that CVAI at baseline was an independent negative predictor of cortical vBMD at month 12 (distal radius; B = − 0.626, *p* = 0.022, 95%CI − 1.914 to − 0.153, tibia: B = − 0.394, *p* = 0.003, 95%CI − 1.366 to − 0.290, Table 3). CVAI was not involved in cortical vBMD of MC2 at month 12 (*p* > 0.05, data not shown). Meanwhile, no significant differences existed between CVAI and cortical vBMD in MC2, distal radius and distal tibia at baseline (all *p* > 0.05, data not shown). A separate analysis using high vs low CVAI subgroup at baseline demonstrated similar findings [High CVAI group had a significantly lower tibial cortical vBMD at Month 12 (Exp [B] = 0.988, 95% CI 0.979–0.998, *p* = 0.019)] (Supplementary Table 5).

**Table 3:** The association between baseline CVAI and bone microstructure at month 12 in female ERA.

	Univariate			Multivariate		
	Beta	*p* value	95%CI	Beta	*p* value	95%CI
Distal radius cortical vBMD at month 12						
CVAI	− 0.262	0.061	− 0.916 to 0.022	− 0.626	0.022	− 1.914 to − 0.153
Age	− 0.543	0.000	− 4.071 to − 1.584			
BMI	0.115	0.115	− 2.968 to 7.074	0.502	0.012	2.221–17.350
Distal tibia cortical vBMD at month 12						
CVAI	− 0.394	0.003	− 1.366 to − 0.290	− 0.394	0.003	− 1.366 to − 0.290
Age	− 0.641	0.000	− 5.471 to − 2.736			
BMI	0.009	0.948	− 5.912 to 6.311			
HBP	− 0.272	0.047	− 2.269 to − 0.017			

vBMD: volumetric bone mineral density; CI: confidence interval; CAVI: Chinese adiposity visceral index; BMI: body mass index; HBP: hypertension; ERA: early rheumatoid arthritis.

In terms of changes in vBMD and microstructure over the period of 12 months, CVAI at baseline was also an independent predictor of the change in trabecular vBMD (tibia: B = 0.444, *p* = 0.001, 95% CI 0.018–0.063; distal radius: B = 0.356, *p* = 0.008, 95% CI 0.403–0.063) (Table 4). There was no significant association between baseline CVAI and HR-pQCT parameters at MC 2 (at month 12 or changes over the period of 12 months) (data not shown).

**Table 4:** Association between CVAI and changed bone microstructure in female ERA.

	Univariate			Multivariate		
	Beta	*p* value	95%CI	Beta	*p* value	95%CI
Decreased trabecular vBMD in distal tibia						
CVAI	0.444	0.001	0.018–0.063	0.444	0.001	0.018–0.063
Age	0.286	0.036	0.018–0.522			
BMI	0.437	0.001	0.051–0.189			
HC	0.262	0.056	− 0.003 to 0.277			
Decreased trabecular vBMD in distal radius						
CVAI	0.289	0.038	0.011–0.360	0.356	0.008	0.403–0.063
Age	0.318	0.021	0.096–1.151			
BMI	− 0.119	0.199	− 2.684 to 1.088			
ACCP	0.281	0.046	0.244–26.229			
RF	0.293	0.035	1.257–33.422	0.333	0.013	4.292–35.053

vBMD: volumetric bone mineral density; CI: confidence interval; CAVI: Chinese adiposity visceral index; HC: hip circumference; BMI: body mass index; ERA: early rheumatoid arthritis; RF: rheumatoid factor; ACCP: Autoantibodies binding to citrullinated antigens.

## Discussions

To the best of our knowledge, this study is a prospective study to explore the associations between visceral dysfunction of fat metabolism (CVAI) and bone microstructure assessed using HR-pQCT in early RA patients. We demonstrated that CVAI was significantly higher in female ERA patients compared with age- and BMI- matched healthy controls, which was associated with several disease severity parameters, indicating that adipose tissue dysfunction may be related to inflammation in RA patients. This finding concurs with previous report suggesting that obesity is associated with higher CRP levels and ESR in women with RA. This association is related to fat mass and not RA disease activity^18^.

Several studies have demonstrated that VAT rather than subcutaneous adipose tissue plays a crucial role in metabolic/inflammation diseases^19,20^. Although the clinical index VAI was significantly related to metabolic diseases^15^, our data did not show any statistical differences in VAI between the ERA group and healthy controls (*p* > 0.05), which suggests that this parameter may not be able to reflect visceral fat dysfunction in these patients. The underlying reason may be related to ethnic variations in body fat distribution^21^, as Asians are characterized by relatively higher body adipose tissue content at a lower BMI comparing with Caucasians, and are more likely to accumulate visceral fat^16^.

Observational studies have identified obesity as a risk factor and a poor prognostic marker of RA, and adipose tissue as a possible source of inflammation^22^. These findings are in line with our results showing that higher inflammatory burden, i.e. reflected by the higher ESR level, RF titer and damaged joint count, were observed in the high CVAI group compared to the low CVAI group, indicating that fat dysfunction may contribute towards the pathophysiology of inflammation in RA. Adipose tissue has been implicated in the development of a low-grade inflammatory state in inflammatory conditions including RA by the production of pro-inflammatory adipocytokines^22^. Dysregulation of immune-endocrine circuits involved in the progression of chronic metabolic disorders (such as obesity) also plays a critical role in chronic inflammatory diseases such as RA^23^. Cytokines secreted by an excess of adipose tissue could have an effect on the whole body, resulting in the progression of a named low-level inflammation, which can be found in obese individuals^24^. Chronic inflammation caused by the adipocytokines may contributes towards irreversible damage of cartilage and bone^25^. Our data provided preliminary data supporting the relationship between visceral adipose tissue dysfunction and deterioration in bone microstructure, including its index vBMD. It is reported that serum and synovial fluid levels of the adipokine fatty acid-binding protein (FABP4) are much higher in patients with RA compared with osteoarthritis (OA)^26^. Levels of FABP4 were positively associated with BMI in patients with RA but did not correlate with disease activity and, until now, there is no evidence that FABP4 affects bone remodeling in RA^25^. Whether FABP4 may be involved in the association between adipose tissue and bone loss in ERA still needs further investigation.

In our study, there was a correlation between CVAI and damaged joint count, increased cortical porosity diameter at the MCP2, as well as a lower cortical vBMD (at the distal tibia at baseline, and at the distal radius/tibia at month 12), suggesting that adipose tissue may adversely affect the bone microstructure. Indeed, preadipocyte differentiation is a complicated procedure depending on hormonal and nutrient adequacy, which involves activation of various transcription factors^27^. Interestingly, adipocytes share the same stem cell precursors with osteoblasts and chondrocytes^28,29^ and under certain conditions their precursors can differentiate to osteoblasts and macrophages^28,30^. Studies show that adipocytes secrete adipokines, cytokines, chemokines and complement factors, which may be involved in the bone metabolism of RA. Adiponectin, a well-studied adipokine, can increase the capacity of osteoblasts to mineralize bone and increase the expression of osteoblast-related genes during osteogenic differentiation, via phosphorylation of AMP-activated protein kinase (AMPK) pathway^31^, suggesting a pro-osteogenic effect of this adipokine during bone remodeling. An increased risk for obese subjects with high serum adiponectin levels at baseline to develop RA, specifically with high adiponectin and CRP levels, was recently described in a study during a follow-up for up to 29 years^32^. Another recent study showed that Baricitinib, a JAK inhibitor blocking central inflammatory signaling pathways decreases systemic inflammation biomarkers, such as IL-6, CRP, as well as adiponectin, in rheumatoid arthritis patients^33^. Leptin, another adipokine, may induce higher expression of vascular cell adhesion protein 1 (VCAM-1) in primary human chondrocytes mediated by Janus kinase 2 (JAK2) and phosphatidylinositol 3-kinase (PI3K) signaling^34^. These central signaling pathways contribute to cellular activation and inflammatory processes. ADAMTS4, ADAMTS5 and ADAMTS9 expression could be induced by leptin in human chondrocytes via the activation of proinflammatory signaling pathways involving mitogen-activated protein kinase (MAPK) and NFκB^35^. Interestingly, high levels of systemic leptin and vaspin were identified in early RA compared to healthy controls^36^. Another study addressed RA patients with long disease duration (≥ 5 years), showing that pro-inflammatory markers, such as TNF-alpha as well as resistin and leptin, were highest in long-duration RA, although they also increased in short-duration RA (< 1 year) compared to healthy controls^37^. These events promote pro-inflammatory cytokine production in joint cartilage, causing chondrocyte apoptosis, metalloproteases activation and consequently inflammation. Nonetheless, the relationship between CVAI, adipocytokine (adiponectin and leptin et al.) and bone microstructure would need further studies in ERA patients.

Our study has several limitations. First, although our study sample size was sufficient to meet the end points by using G-power analysis, this cohort study was still relatively small, consisting of convenience sample of female, and thus the results could have been influenced by selection bias. Large sample size and male should be enrolled in the future and confirm our results. Second, we did not measure the adipocytokine in our study, which refers to all factors produced by adipocytes, including adipokines, cytokines, chemokines and complement factors. Therefore, we did not know the relationship between adipocytokine and bone microstructure, and we may explore its association in the future study. Third, due to the study, we may not indicate the cause and effect of CVAI on bone microstructure among all the ERA patients. Fourth, because all the patients in this study were Southern Chinese, our results may not be extrapolated in other ethnicities. Fourth, a clear limitation of HR-pQCT-based evaluation of the cortical pore network is that HR-pQCT cannot resolve cortical porosity at the level of the smallest Haversian and Volkmann canals. However, the power of HR-pQCT indices of cortical porosity to discriminate fracture status, menopausal status, race, and age have demonstrated the practical utility of evaluating resolvable microstructure. Additionally, in cohorts experiencing pathologic porosity increases, pore sizes are considerably larger than the resolution limit of HR-pQCT^38^. Fifth, the content of patients’ physical activity or exercise was not recorded in our study. In the future, physical activity or exercise should be included to evaluate its role in ERA patients. Meanwhile, the MC2, distal tibia and distal radius should also be scanned by HR-p QCT in female healthy control in the future. Last but not the least, we did not measure the sexual hormones in female ERA patients, and not sure whether they may also be an important bio-factor contributing to the association between CVAI and bone microstructure.

## Conclusions

The current clinical follow-up study suggests that CVAI is an independent predictor of trabecular bone deterioration in female ERA patients.

## Methods

### Participants

This was one of a group of studies, open-labelled, randomized controlled trials (RCT) in Hong Kong applying for exploring the role of two intense control treatment approaches on vascular stiffness in early RA patients (clinicaltrial.gov no: NCT01768923,16/01/2013)^39^. One hundred and four female ERA patients in this current study were all chosen from this RCT. This study mainly focused on the association between CVAI and bone microstructure detected by HR‐pQCT. In other words, 104 consecutive ambulatory Chinese female ERA patients with symptom duration below 24 months were enrolled from the department of rheumatology in the Prince of Wales Hospital and other hospitals in Hong Kong. Participants were recruited once they fulfilled the American College of Rheumatology (ACR)/EULAR revised criteria for RA (2010 version)^40^. Participants were excluded if they: (1) had a lower creatinine clearance (< 30 ml/min) or cancer treated by chemistry or radiology; (2) were on antiosteoporosis medication, including oral bisphosphonates, teriparatide, double acting bone preparation, hormone replacement therapy, or RANKL inhibitor or bDMARDs; (3) had critical deformities at the metacarpophalangeal joints which may prevent a credible HR-pQCT scan leading to the motion artefacts with inaccurate scan; (4) were pregnant or lactation. All patients were given medical strategies based on a standard method with the purpose of the remission for 1 year^41^. One hundred age-, BMI- and gender-matched apparently healthy female volunteers without rheumatic diseases and bone diseases were enrolled in the ward of mouth recommendation department at the Prince of Wales hospital. The study was launched following the Declaration of Helsinki and established by the Clinical Research Ethics Committee [Joint Chinese University of Hong Kong‐New Territories East Cluster (CRE 2011.483‐T)]. All the patients and volunteers provided by writing consent forms.

### HR-pQCT imaging and image evaluation

The methodology information has been reported before^42,43^. In other words, distal radius and tibia in the non-dominant side of the patients, and the second metacarpal head (MC2) of the non-dominant hand of the patients were explored by the first-generation HR-pQCT (XtremeCT I, Scanco Medical AG, Bruttisellen, Switzerland) at baseline and 12 months^43^. HR‐pQCT was operated by an experienced practitioner (VWH) who was not aware of any clinical data of the patients. At the same time, she conducts the daily and weekly calibration according to the protocols, which is extremely important for maintaining high standards of reproducibility as well as reliability and low variability in the short and long term. All the images were assessed by one professional performer who evaluated motion artifact followed by a mature system published by Pialat and his colleagues, which graded images based on different situation named grade 1 to grade 5^44^. Images were assessed as grade 1 or 2 if there was no or minor motion artifact. If there was severe or extreme motion artifact in these participants, they would be excluded from further study and be considered to unsuccessful scan.

The whole interested volume was divided into cortical and trabecular components by applying a completely automated strategy named cortical compartment segmentation methodology by manual corrections with good short-term and long-term reproducibility^45,46^. 3D registration was applied here. Recently, the 3D registration improved the identification of the common region retained for longitudinal analysis, contributing to improve the reproducibility of cortical bone parameter measurements in HR-pQCT^47^.

### Clinical interview, anthropometrical and serum laboratory measurements

Clinical parameters assessed included the extra-articular manifestations, tender and swollen joint count, damaged joints’ number, and the modified health assessment questionnaire of those patients (HAQ). Previous medical strategies were inquired and recorded when patient was interviewed^48^. Standardized medical data collection documents was applied when these patients were interviewed and explored.

Body height and weight were evaluated without shoes and outer clothing. BMI was explored following the formula: weight in kilograms divided by square of height in meters. WC was assessed by using a soft tape at midway between the lowest rib and iliac crest in standardized standing situation.

All the blood samples were taken at fasting status for at least 10 hours. RF and ACCP were evaluated at baseline. Other tests including complete blood count, plasma FBG, serum TC, HDL-c, TG, LDL-c, ESR, CRP, liver and renal function was assessed every 3 months.

### Definition of visceral obesity, obesity and calculation of CVAI as well as VAI

Based on 2006 World Health Organization (WHO), the criterion for central obesity is that waist circumference (WC) is above 80 cm for females, and the criterion for Chinese overweight/obesity is that BMI is above 24 kg/m^2^^49^. CVAI for female was measured by the following formula: CVAI = − 187.32+1.71× age + 4.23 × BMI +1.12 × WC (cm) + 39.76 × Log10 TG − 11.66 × HDL-C. Visceral adiposity index (VAI) was assessed by applying the formula established in Caucasians previously^11^. VAI = (WC/39.58 + (1.89 × BMI)) × (TG/0.81) × (1.52/HDL).

### Statistical analysis

All statistical analyses were conducted by applying SPSS Statistics Version 22 (SPSS, Chicago, IL). The data were expressed as mean ± Standard Deviation (SD), except for skewed variables, which were shown by median and interquartile range (25–75%) given in parentheses. For continuous parameters, the differences between both groups were calculated by either Mann‐Whitney U test or Student's *t* test, whereas the Chi-squared test was used for comparisons in terms of categorical parameters between two groups. The relationships between levels of CVAI and variables of cortical/trabecular bone density, microstructure, and parameters associated with disease activity/severity were explored by applying Spearman correlation. Linear or logistic regression analysis was conducted to evaluate the relationship between CVAI at baseline and bone microstructure (at distal radius, distal tibia and MC2) after adjusting for other confounding variables at month 12 as well as the change over a period of 12 months. Potential explanatory variables included in the regression analyses were shown in Tables 1 and 2. Parameters with a *P* value of < 0.1 in the univariate analysis were recruited in the multivariate analysis. For all the analyses, a two tailed *p* ≤ 0.05 was recognized as indicating statistical significance.

### Ethical approval and consent to participate

The study was approved by the Clinical Research Ethics Committee [Joint Chinese University of Hong Kong‐New Territories East Cluster (CRE 2011.483‐T)]. Written informed consents were obtained from all patients according to the Declaration of Helsinki. Signed informed consent was obtained from every study participant in accordance with the Helsinki Declaration.

## Supplementary Information

Supplementary Tables.

## Data Availability

The datasets generated and/or analysed during the current study are not publicly available due to no publication yet but are available from the corresponding author on reasonable request.

## Abbreviations

ERA: Early rheumatoid arthritis
CVAI: Chinese visceral adiposity index
vBMD: Cortical volumetric bone mineral density
BMD: Bone mineral density
DXA: Dual-energy x-ray absorptiometry
HR-pQCT: High-resolution peripheral quantitative computed tomography
WAT: White adipose tissue
VAT: Visceral adipose tissue
CT: Computed tomography
MRI: Magnetic resonance imaging
VAI: Visceral adiposity index
WC: Waist circumference
BMI: Body mass index
TG: Serum triglycerides
HDL: High-density lipoprotein
MAFLD: Metabolic fatty liver disease
NASH: Non-alcoholic steatohepatitis
RCT: Randomized controlled trials
MC2: Second metacarpal head
RF: Rheumatoid factor
ACCP: Anti-cyclic citrullinated peptide antibody
LDL-c: Low-density lipoprotein-cholesterol
ESR: Erythrocyte sedimentation rate
AMPK: AMP-activated protein kinase
CRP: C-response protein
VCAM-1: Vascular cell adhesion protein 1
