# Supplementary material for: A novel visceral adiposity index predicts bone loss in female early rheumatoid arthritis patients detected by HR-pQCT
Source: Sci Rep. 2023 Feb 11;13:2471. doi: 10.1038/s41598-023-29505-z (PMC9922327; doi:10.1038/s41598-023-29505-z)
Supplement: Supplementary file 1 — Supplementary Tables. [file 41598_2023_29505_MOESM1_ESM.docx]

Supplementary Table 1. The bone microstructure of MC2, distal tibia and distal radius in female ERA at baseline and month 12.

|  | **At baseline** | **Month 12** | ***P* value** |
| --- | --- | --- | --- |
| **MC2** |  |  |  |
| Tot Area (mm^2^) | 93.64±8.00 | 93.23±9.22 | 0.043 |
| Cort Area (mm^2^) | 6.29±4.16 | 6.19±4.15 | 0.035 |
| Trab Area (mm^2^) | 77.00±7.32 | 76.79±8.30 | 0.041 |
| D100 (mg HA/cm^3^) | 267.34±52.63 | 265.88±49.73 | 0.692 |
| Dcomp (mg HA/cm^3^) | 515.81±91.51 | 511.82±84.55 | 0.528 |
| Ct.Th (mm) | 0.16±0.10 | 0.32±1.30 | 0.361 |
| Ct.Pm (mm) | 39.80±1.69 | 40.14±2.72 | 0.202 |
| Dtrab (mg HA/cm^3^) | 204.68±39.72 | 201.24±46.19 | 0.374 |
| Dmeta (mg HA/cm^3^) | 226.74±92.18 | 235.69±50.60 | 0.499 |
| Dinn (mg HA/cm^3^) | 169.10±83.63 | 177.45±43.16 | 0.476 |
| Meta/Inn (%) | 1.34±0.16 | 1.67±2.29 | 0.300 |
| BV/TV (%) | 0.17±0.034 | 0.17±0.033 | 0.898 |
| Tb.N (1/mm) | 1.82±0.27 | 2.13±2.20 | 0.310 |
| Tb.Th (mm) | 0.094±0.016 | 0.094±0.017 | 0.898 |
| Tb.Sp (mm) | 0.47±0.085 | 0.47±0.083 | 0.617 |
| Tb.1N.SD (mm)  **Distal Tibia** | 0.29±0.095 | 0.21±0.54 | 0.271 |
| Tot Area (mm^2^) | 614.00±94.40 | 593.19±81.78 | 0.027 |
| Cort Area (mm^2^) | 101.59±22.76 | 101.00±22.00 | 0.048 |
| Trab Area (mm^2^) | 505.48±91.49 | 485.78±79.29 | 0.032 |
| D100 (mg HA/cm^3^) | 261.05±57.23 | 262.43±53.49 | 0.830 |
| Dcomp (mg HA/cm^3^) | 859.79±72.68 | 860.75±72.92 | 0.893 |
| Ct.Th (mm) | 1.05±0.23 | 1.04±0.23 | 0.913 |
| Ct.Pm (mm) | 97.27±7.43 | 95.52±6.37 | 0.021 |
| Dtrab (mg HA/cm^3^) | 128.72±39.57 | 128.74±38.63 | 0.998 |
| Dmeta (mg HA/cm^3^) | 196.61±42.08 | 197.01±40.42 | 0.942 |
| Dinn (mg HA/cm^3^) | 82.55±39.54 | 82.26±38.60 | 0.953 |
| Meta/Inn (%) | 3.60±4.68 | 3.26±3.30 | 0.244 |
| BV/TV (%) | 0.11±0.033 | 0.11±0.032 | 0.997 |
| Tb.N (1/mm) | 1.41±0.33 | 1.44±0.30 | 0.506 |
| Tb.Th (mm) | 0.076±0.012 | 0.074±0.013 | 0.274 |
| Tb.Sp (mm) | 0.68±0.22 | 0.66±0.19 | 0.290 |
| Tb.1N.SD (mm)  **Distal radius** | 0.37±0.26 | 0.35±0.20 | 0.250 |
| Tot Area (mm^2^) | 220.33±44.94 | 207.00±30.26 | 0.018 |
| Cort Area (mm^2^) | 51.85±13.71 | 50.31±11.87 | 0.047 |
| Trab Area (mm^2^) | 164.00±40.77 | 152.97±28.57 | 0.033 |
| D100 (mg HA/cm^3^) | 325.57±77.46 | 326.61±77.34 | 0.909 |
| Dcomp (mg HA/cm^3^) | 886.10±69.05 | 905.94±60.20 | 0.012 |
| Ct.Th (mm) | 0.83±0.21 | 0.92±0.63 | 0.311 |
| Ct.Pm (mm) | 62.57±6.53 | 59.49±8.48 | 0.011 |
| Dtrab (mg HA/cm^3^) | 126.02±44.45 | 114.86±46.10 | 0.089 |
| Dmeta (mg HA/cm^3^) | 186.95±44.61 | 173.46±49.73 | 0.047 |
| Dinn (mg HA/cm^3^) | 83.67±45.99 | 74.05±45.84 | 0.145 |
| Meta/Inn (%) | 2.75±1.78 | 3.32±4.16 | 0.143 |
| BV/TV (%) | 0.10±0.037 | 0.06±0.293 | 0.250 |
| Tb.N (1/mm) | 1.42±0.32 | 1.31±0.48 | 0.105 |
| Tb.Th (mm) | 0.073±0.015 | 0.082±0.089 | 0.449 |
| Tb.Sp (mm)  Tb.1N.SD (mm) | 0.69±0.35  0.32±0.28 | 0.66±0.47  0.30±0.23 | 0.551  0.285 |

MC: Metacarpal head ; ERA: early rheumatoid arthritis; Cort: Cortical; Trab: Trabecular; BMD: bone mineral density; D100: average BMD; Dcomp: compact BMD; Ct.Th: cortical thickness; Ct.Pm: cortical porosity diameter; Dtrab: trabecular BMD; Dmeta: meta trabecular BMD; Dinn: inner trabecular BMD; Meta/inn: ratio meta to inner density; BV/TV: trabecular bone volume fraction; Tb.N: number of trabeculae; Tb.Th: trabecular thickness; Tb.Sp: trabecular separation; Tb.1/N.SD: inhomogeneity of network. *P* value: at baseline & Month 12.

Supplementary Table 2. The bone microstructure of MC2 in female ERA based on CVAI.

|  | **low CVAI (n=52)** | **high CVAI (n=52)** | ***P* value** |
| --- | --- | --- | --- |
| Tot Area (mm^2^) | 93.66±7.27 | 94.62±9.45 | 0.596 |
| Cort Area (mm^2^) | 6.16±4.13 | 6.42±4.51 | 0.779 |
| Trab Area (mm^2^) | 75.34±6.92 | 75.75±8.76 | 0.810 |
| D100 (mg HA/cm^3^) | 268.48±54.67 | 262.86±57.29 | 0.639 |
| Dcomp (mg HA/cm^3^) | 513.26±93.92 | 512.10±98.00 | 0.955 |
| Ct.Th (mm) | 0.16±0.11 | 0.16±0.11 | 0.943 |
| Ct.Pm (mm) | 39.39±1.76 | 40.32±1.98 | 0.021 |
| Dtrab (mg HA/cm^3^) | 207.60±40.99 | 199.04±41.92 | 0.335 |
| Dmeta (mg HA/cm^3^) | 224.18±101.91 | 231.76±46.67 | 0.652 |
| Dinn (mg HA/cm^3^) | 171.24±91.58 | 175.96±40.24 | 0.753 |
| Meta/Inn (%) | 1.30±0.15 | 1.34±0.15 | 0.283 |
| BV/TV (%) | 0.17±0.034 | 0.17±0.035 | 0.342 |
| Tb.N (1/mm) | 1.80±0.28 | 1.81±0.26 | 0.948 |
| Tb.Th (mm) | 0.097±0.019 | 0.009±0.018 | 0.243 |
| Tb.Sp (mm) | 0.47±0.085 | 0.47±0.080 | 0.938 |
| Tb.1N.SD (mm) | 0.28±0.078 | 0.30±0.10 | 0.370 |
| Changed Cort Area (mm^2^) | -0.18±0.61 | -0.63±2.83 | 0.074 |
| Changed Trab Area (mm^2^) | 1.37±2.21 | 0.91±5.03 | 0.087 |
| Changed D100 (mg HA/cm^3^) | 1.89±12.85 | -4.45±34.71 | 0.170 |
| Changed Dcomp (mg HA/cm^3^) | 2.63±24.49 | -0.98±58.31 | 0.128 |
| Changed Ct.Th (mm) | 0.37±1.91 | -0.019±0.068 | 0.084 |
| Changed Ct.Pm (mm) | 0.028±2.40 | 0.62±1.32 | 0.655 |
| Changed Dtrab (mg HA/cm^3^) | -4.04±30.58 | -2.90±22.54 | 0.533 |
| Changed Dmeta (mg HA/cm^3^) | 20.96±136.34 | -1.77±29.80 | 0.345 |
| Changed Dinn (mg HA/cm^3^) | 21.91±121.59 | -3.76±19.05 | 0.301 |
| Changed Tb.N (1/mm) | 0.65±3.19 | 0.004±0.16 | 0.824 |
| Changed Tb.Th (mm) | 0.046±0.23 | 0.0003±0.0098 | 0.675 |
| Changed Tb.Sp (mm) | 0.63±3.16 | 0.0043±0.047 | 0.796 |
| Changed Tb.1N.SD (mm) | -0.16±0.77 | -0.0083±0.063 | 0.575 |

MC: Metacarpal head ; CVAI: Chinese visceral adiposity index; ERA: early rheumatoid arthritis; Cort: Cortical; Trab: Trabecular; BMD: bone mineral density; D100: average BMD; Dcomp: compact BMD; Ct.Th: cortical thickness; Ct.Pm: cortical porosity diameter; Dtrab: trabecular BMD; Dmeta: meta trabecular BMD; Dinn: inner trabecular BMD; Meta/inn: ratio meta to inner density; BV/TV: trabecular bone volume fraction;Tb.N: number of trabeculae; Tb.Th: trabecular thickness; Tb.Sp: trabecular separation; Tb.1/N.SD: inhomogeneity of network. *P* value: low CVAI & high CVAI.

Supplementary Table 3. The bone microstructure of distal tibia in female ERA based on CVAI.

|  | **low CVAI (n=52)** | **high CVAI (n=52)** | ***P*value** |
| --- | --- | --- | --- |
| Tot Area (mm^2^) | 618.31±96.80 | 593.82±75.28 | 0.185 |
| Cort Area (mm^2^) | 100.38±21.99 | 100.18±24.64 | 0.967 |
| Trab Area (mm^2^) | 511.73±91.89 | 486.64±76.58 | 0.164 |
| D100 (mg HA/cm^3^) | 261.61±57.17 | 264.28±55.55 | 0.824 |
| Dcomp (mg HA/cm^3^) | 869.67±71.85 | 823.72±72.19 | 0.024 |
| Ct.Th (mm) | 1.03±0.22 | 1.05±0.26 | 0.751 |
| Ct.Pm (mm) | 97.47±7.66 | 95.82±6.01 | 0.260 |
| Dtrab (mg HA/cm^3^) | 131.77±39.79 | 132.50±35.79 | 0.927 |
| Dmeta (mg HA/cm^3^) | 199.21±42.16 | 204.78±38.75 | 0.518 |
| Dinn (mg HA/cm^3^) | 85.90±39.60 | 83.34±36.31 | 0.752 |
| Meta/Inn (%) | 3.12±3.07 | 3.47±4.32 | 0.658 |
| BV/TV (%) | 0.11±0.033 | 0.11±0.030 | 0.929 |
| Tb.N (1/mm) | 1.45±0.31 | 1.41±0.29 | 0.465 |
| Tb.Th (mm) | 0.075±0.012 | 0.079±0.014 | 0.190 |
| Tb.Sp (mm) | 0.65±0.21 | 0.67±0.18 | 0.716 |
| Tb.1N.SD (mm) | 0.34±0.23 | 0.37±0.21 | 0.643 |
| Changed Cort Area (mm^2^) | 1.87±23.70 | -5.58±13.82 | 0.064 |
| Changed Trab Area (mm^2^) | -33.02±65.87 | -5.46±64.31 | 0.017 |
| Changed D100 (mg HA/cm^3^) | 10.19±60.86 | -6.33±30.49 | 0.042 |
| Changed Dcomp (mg HA/cm^3^) | 19.28±57.09 | -15.11±42.76 | 0.006 |
| Changed Ct.Th (mm) | 0.046±0.22 | -0.046±0.14 | 0.016 |
| Changed Ct.Pm (mm) | -2.75±5.43 | -0.91±5.40 | 0.123 |
| Changed Dtrab (mg HA/cm^3^) | -0.036±51.81 | 0.11±19.89 | 0.776 |
| Changed Dmeta (mg HA/cm^3^) | 2.18±55.71 | -1.14±20.96 | 0.581 |
| Changed Dinn (mg HA/cm^3^) | -1.60±49.24 | 0.93±21.69 | 0.943 |
| Changed Tb.N (1/mm) | 0.046±0.41 | 0.015±0.23 | 0.454 |
| Changed Tb.Th (mm) | -0.0019±0.011 | -0.0006±0.011 | 0.708 |
| Changed Tb.Sp (mm) | -0.018±0.18 | -0.052±0.19 | 0.755 |
| Changed Tb.1N.SD (mm) | -0.0064±0.13 | -0.051±0.18 | 0.735 |

CVAI: Chinese visceral adiposity index; ERA: early rheumatoid arthritis; Cort: Cortical; Trab: Trabecular; BMD: bone mineral density; D100: average BMD; Dcomp: compact BMD; Ct.Th: cortical thickness; Ct.Pm: cortical porosity diameter; Dtrab: trabecular BMD; Dmeta: meta trabecular BMD; Dinn: inner trabecular BMD; Meta/inn: ratio meta to inner density; BV/TV: trabecular bone volume fraction;Tb.N: number of trabeculae; Tb.Th: trabecular thickness; Tb.Sp: trabecular separation; Tb.1/N.SD: inhomogeneity of network. *P* value: low CVAI & high CVAI.

Supplementary Table 4. The bone microstructure of distal radius in female ERA based on CVAI.

|  | | **low CVAI (n=52)** | | **high CVAI (n=52)** | | ***P* value** | |
| --- | --- | --- | --- | --- | --- | --- | --- |
| Tot Area (mm^2^) | | 216.60±45.05 | | 216.94±37.38 | | 0.969 | |
| Cort Area (mm^2^) | | 51.79±10.93 | | 49.35±13.90 | | 0.363 | |
| Trab Area (mm^2^) | | 161.10±41.77 | | 162.82±35.84 | | 0.822 | |
| D100 (mg HA/cm^3^) | | 330.75±72.07 | | 316.37±79.93 | | 0.377 | |
| Dcomp (mg HA/cm^3^) | | 900.14±63.03 | | 833.32±73.38 | | 0.011 | |
| Ct.Th (mm) | | 0.85±0.17 | | 0.80±0.22 | | 0.280 | |
| Ct.Pm (mm) | | 61.63±6.57 | | 61.97±5.45 | | 0.788 | |
| Dtrab (mg HA/cm^3^) | | 126.21±42.75 | | 122.87±41.36 | | 0.709 | |
| Dmeta (mg HA/cm^3^) | | 187.70±41.84 | | 183.73±40.52 | | 0.651 | |
| Dinn (mg HA/cm^3^) | | 83.49±44.28 | | 80.58±44.23 | | 0.758 | |
| Meta/Inn (%) | | 2.44±1.27 | | 3.20±3.39 | | 0.174 | |
| BV/TV (%) | | 0.11±0.036 | | 0.10±0.034 | | 0.706 | |
| Tb.N (1/mm) | | 1.42±0.34 | | 1.42±0.34 | | 0.972 | |
| Tb.Th (mm) | | 0.073±0.014 | | 0.072±0.017 | | 0.693 | |
| Tb.Sp (mm) | | 0.71±0.40 | | 0.69±0.31 | | 0.850 | |
| Tb.1N.SD (mm) | | 0.34±0.33 | | 0.34±0.33 | | 0.944 | |
| Changed Cort Area (mm^2^) | | 0.84±12.79 | | -6.81±15.67 | | 0.079 | |
| Changed Trab Area (mm^2^) | | -18.08±45.76 | | -15.88±48.24 | | 0.139 | |
| Changed D100 (mg HA/cm^3^) | | 19.20±78.20 | | -37.48±91.25 | | 0.036 | |
| Changed Dcomp (mg HA/cm^3^) | | 39.04±61.94 | | -62.36±241.56 | | 0.014 | |
| Changed Ct.Th (mm) | | 0.22±0.88 | | -0.09±0.24 | | 0.093 | |
| Changed Ct.Pm (mm) | | -4.78±11.01 | | -5.78±16.12 | | 0.562 | |
| Changed Dtrab (mg HA/cm^3^) | | -10.24±60.93 | | -19.30±38.85 | | 0.014 | |
| Changed Dmeta (mg HA/cm^3^) | | -14.99±64.15 | | -23.61±49.02 | | 0.036 | |
| Changed Dinn (mg HA/cm^3^) | | -7.08±60.17 | | -16.33±35.24 | | 0.035 | |
| Changed Tb.N (1/mm) | | -0.15±0.62 | | -0.15±0.39 | | 0.254 | |
| Changed Tb.Th (mm) | | 0.026±0.13 | | -0.0082±0.020 | | 0.061 | |
| Changed Tb.Sp (mm) | | -0.11±0.55 | | -0.013±0.21 | | 0.226 | |
| Changed Tb.1N.SD (mm) | | -0.055±0.21 | | -0.013±0.099 | | 0.368 | |

CVAI: Chinese visceral adiposity index; ERA: early rheumatoid arthritis; Cort: Cortical; Trab: Trabecular; BMD: bone mineral density; D100: average BMD; Dcomp: compact BMD; Ct.Th: cortical thickness; Ct.Pm: cortical porosity diameter; Dtrab: trabecular BMD; Dmeta: meta trabecular BMD; Dinn: inner trabecular BMD; Meta/inn: ratio meta to inner density; BV/TV: trabecular bone volume fraction; Tb.N: number of trabeculae; Tb.Th: trabecular thickness; Tb.Sp: trabecular separation; Tb.1/N.SD: inhomogeneity of network. *P* value: low CVAI & high CVAI.

Supplementary table 5 Analysis of the correlation between baseline CVAI and bone microstructure at month 12 in female ERA

| **High CVAI at baseline** | | | | | | | | |  |
| --- | --- | --- | --- | --- | --- | --- | --- | --- | --- |
|  | Univariate | | | | Multivariate | | | | |
|  | Exp (B) | *p*value | 95% CI | Exp (B) | | *p* value | 95% CI |  |  |
| distal tibial vBMD | 0.988 | 0.019 | 0.979～0.998 | 0.988 | | 0.019 | 0.979～0.998 |  |  |
| distal radius vBMD | 0.987 | 0.022 | 0.977～0.998 |  | |  |  |  |  |
| MCP 2 vBMD | 0.999 | 0.190 | 0.990～1.008 |  | |  |  |  |  |

CVAI: Chinese visceral adiposity index; ERA: early rheumatoid arthritis; vBMD: volumetric BMD; CI: confidence interval; MCP: Metacarpophalangeal.
